# Intranasal esketamine for ECT-refractory bipolar depression: a case report

**DOI:** 10.3389/fpsyt.2026.1890002

**Published:** 2026-07-30

**Authors:** Muhammad Abbas, Bruno Kallas, Max Garris

**Affiliations:** 1 Hackensack Meridian Raritan Bay Medical Center, Perth Amboy, NJ, United States; 2 Department of Medical Sciences, Hackensack Meridian School of Medicine, Hackensack Meridian Health, Nutley, NJ, United States

**Keywords:** bipolar depression, ECT, esketamine, ketamine, treatment resistant depression

## Abstract

This is a case report of a 52 year old female with ECT-refractory bipolar depression who was treated with esketamine nasal spray, leading to a noticeable improvement in her depressive symptoms. She experienced a relapse after a reduction in her maintenance treatment in conjunction with psychosocial stressors, but improved once again after returning to her induction regimen. She tolerated the treatment well with minimal side effects and no reported manic or hypomanic events. More robust research is warranted to better characterize the effectiveness of esketamine in treating treatment-resistant bipolar depression.

## Introduction

Bipolar disorder affects roughly 8 million adults in the United States and about 40 million people globally (1). The clinical weight of this disorder is primarily driven by the depressive phase, given that patients suffer through significantly longer periods of depressive symptoms compared to manic or hypomanic states (2, 3). Individuals with bipolar disorder have a 20 to 30 times higher risk of suicide than the general population, largely due to the burden of depressive symptoms (3).

Specific attention has recently been directed toward developing therapies for treatment-resistant bipolar depression (TRBPD), a condition with no FDA-approved treatment (4). According to the International Society for Bipolar Disorders, TRBPD is the failure to achieve a significant and sustained clinical response after at least two approved and adequately dosed pharmacological treatments, administered for a sufficient duration with treatment adherence (5). For bipolar I depression, approved treatments include quetiapine (300–600 mg/day for ≥ 8 weeks), lurasidone (20–120 mg/day for ≥ 6 weeks), the combination of olanzapine (6–12 mg/day) and fluoxetine (25–75 mg/day for ≥ 8 weeks), cariprazine (1.5–3 mg/day for ≥ 6 weeks), and lumateperone (42 mg/day for ≥ 6 weeks). For bipolar II depression, approved treatments include quetiapine (300–600 mg/day for ≥ 8 weeks) and lumateperone (42 mg/day for ≥ 6 weeks) (5). While not FDA-approved for acute bipolar depression, lithium, aripiprazole, and lamotrigine are indicated for maintenance therapy in bipolar disorder (6).

In the last decade, ketamine and esketamine, both NMDA receptor antagonists, have been utilized therapeutically for treatment-resistant depression (TRD). Esketamine, in particular, can be routinely delivered intranasally in an outpatient setting to TRD patients regardless of psychiatric comorbidity, with transient side effects that are rarely severe, and minimal addictive risk (7). The most common transient side effects are dissociation, dizziness, and increased blood pressure (8).

Ketamine and esketamine have also shown promise across multiple studies in the treatment of bipolar depression (9–12). Research to date has displayed that both drugs can reduce suicidal ideation within a day after administration, and are well-tolerated with low risk for manic or hypomanic switch (11–14). Only a handful of hypomanic switches have been documented, many of them being patients who received ketamine or esketamine alongside another antidepressant treatment (10). Results from a naturalistic study by Martinotti et al. showed that patients with TRBPD had comparable levels of improvement in their depression with no significant difference in affective switch rates in relation to their TRD counterparts when treated with esketamine. Additionally, the study found esketamine to have a greater anxiolytic effect in TRBPD patients, suggesting that the treatment can also be used to alleviate comorbid anxiety (12).

Results from phase III trials have shown esketamine’s effectiveness in improving depressive symptoms in patients with unipolar depression (15, 16). Nevertheless, the FDA has yet to conduct clinical trials with respect to esketamine for bipolar depression, a reflection of the novelty of this field and the need for further research.

This case report is about a 52 year old female with TRBPD (bipolar 1 subtype) who went into remission for 3 months after receiving intranasal esketamine. This report adds to the growing esketamine literature by showing that an ECT-refractory bipolar disorder patient with predominantly depressive symptoms can experience rapid benefit following esketamine.

## Case presentation

A 52 year old female with a 10 year history of TRBPD presented in 2025 with one month of worsening depressive symptoms, including anhedonia, difficulty concentrating, lack of motivation, sleep disturbances, and passive suicidal ideation without intent. She was first diagnosed with Bipolar Type 1 at age 42, with multiple hospitalizations for depressive episodes over the past decade. Her previous history was largely depressive, with only one noted manic episode occurring at the time she was first diagnosed with Bipolar Disorder. Her treatment history includes multiple classes of antidepressants and adjuvant psychotherapy, with minimal to moderate benefit. This includes SSRIs, atypical antidepressants, mood stabilizers, and anti-psychotics. Her past medical history is also significant for anxiety, insomnia, and hypothyroidism. At the time of admission, she also endorsed social stressors including her son’s incarceration, declining health of her spouse, and personal legal troubles.

Based on current depression symptoms and lack of clinical benefit from past treatments, the patient initiated electroconvulsive therapy (ECT) in March 2025. Written informed consent was obtained for ECT, and separate consent for use of anonymized clinical information. She continued psychotropic medications lamotrigine, risperidone, and trazodone through the course of ECT treatment.

**Figure 1:**
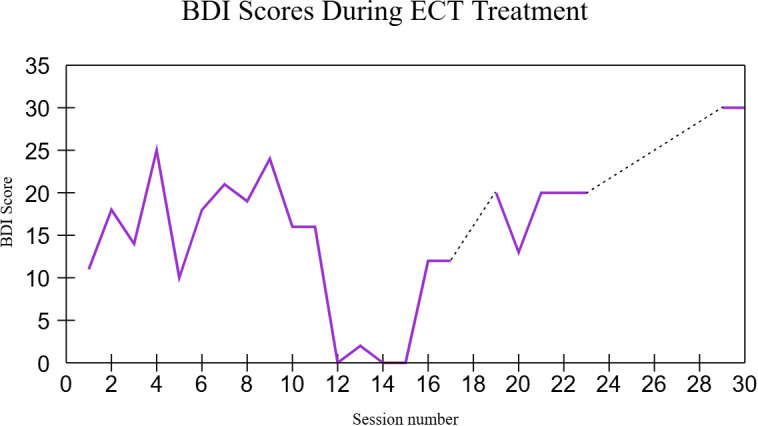
Beck Depression Inventory (BDI) scores following each session of ECT treatment. BDI scores range from 0-63, with higher scores indicating more severe depressive symptoms. The patient refused BDI during sessions 18 and 23-28 as indicated by the dotted line.

General anesthesia with methohexital sodium, succinylcholine, and ketamine was well-tolerated throughout the duration of ECT treatment. ECT stimulus parameters were within standard therapeutic measures, and adequate EEG seizure durations were consistently generated. Beck Depression Inventory (BDI) scores were intended to be recorded after each session; however, they were refused after a number of the later sessions. As shown in **Figure 1**, highest BDI score during ECT treatment was 30, and lowest BDI score achieved during treatment was 0. BDI score trended downwards until July, where it remained over 20 for the rest of the sessions. The five initial ECT sessions used right unilateral (RUL) placement, this was switched to bifrontal placement for the remainder of the sessions. Frequency of sessions was initially started at 3 times weekly, then decreased to once weekly in May 2025. Frequency was then again increased to 3 times weekly in June 2025, continuing until the end of ECT treatment. She had an Emergency Department (ED) admission on 08/04/25, and ECT was subsequently discontinued on 08/18/25 due to subtherapeutic outcomes. At the time of her last treatment, she had completed 30 ECT sessions.

Intranasal esketamine was introduced on 09/19/25 at initial dosing of 56 mg twice weekly. The patient was educated on risks and side effects from esketamine prior to first treatment, with REMS forms completed through the duration of treatment. Esketamine was administered in-office at the Raritan Bay Medical Center’s Neuromodulation Clinic. The patient was also taking aripiprazole daily at the time of treatment. While esketamine nasal spray is currently only indicated as monotherapy or in conjunction with oral antidepressants, adjunctive aripiprazole was determined to be appropriate by the supervising physician. Before initial treatment, she presented with flat but pleasant affect. She experienced nausea and vomiting after receiving esketamine, and was reminded to fast prior to treatment. Frequency was adjusted to once per week starting 10/17/25, then every two weeks starting 11/20/25. She did not experience side effects at any sessions other than the initial session. She endorsed good response to esketamine therapy, with BDI scores remaining less than 5 from initial session to mid-December. She also related improvements in sleep duration and cognitive clarity.

The patient experienced reemergence of depressive symptoms in December 2025, including passive suicidal ideations, prompting self-admission to the ED. Although she experienced a relapse in depressive symptoms, she expressed a desire to continue esketamine treatment. She attributed the relapse in symptoms to skipping doses of her aripiprazole and interpersonal stressors, including verbal conflict with family members and her husband’s worsening health. Following the ED admission, her medication was changed from aripiprazole to lithium. Additionally, esketamine dosage was briefly changed to 84 mg for her first 3 sessions upon restarting. Follow up in the next few weeks showed a marked downward trend in depressive symptoms as well as BDI/PHQ-9 scores. The patient also endorsed “very good” mood and related that she felt that she could return to working soon. As the patient is continuing treatment, ongoing follow-up will allow assessment of longer-term treatment outcomes.

## Discussion

This case highlights the potential benefit of esketamine therapy in patients with bipolar depression who were previously nonresponsive to antipsychotic or ECT therapy. A rapid improvement in this patient’s depressive symptoms was observed following the initiation of esketamine – as noted by a PHQ-9 score of 0 after her second session – with no notable side effects. Her depression remitted for 3 months until a hospitalization in December 2025, which is believed to have been largely driven by psychosocial stressors. Even after being admitted, the patient reported that her depression had improved since starting esketamine and was agreeable to continue her treatment. As depicted in **Table 1**, her most recent treatment visits show lingering depressive symptoms, but were generally downtrending from her relapse. Although her initial results are promising, any optimism should be taken with caution. The long term impact of esketamine on this patient’s bipolar depression is still unclear, as is the extent to which psychosocial stressors could interfere with her outcomes. It is also worth noting that the patient’s relapse only occurred after her esketamine regimen was reduced to once every two weeks in November 2025, which raises questions about the ideal dose and frequency of administration to sustain her remittance. Continued follow-up will elicit more information about the safety and effectiveness of esketamine for this patient.

**Table 1:** Recorded beck depression inventory (BDI) and patient health questionnaire 9 (PHQ-9) scores following esketamine treatment.

Esketamine session number	Dose size (mg)	BDI score	PHQ-9 score
2	56	N/A	0
4	56	0	N/A
6	56	N/A	0
8	56	0	N/A
11	56	0	N/A
12	56	N/A	0
13	56	0	0
14	56	N/A	0
15	56	5	N/A
16	56	18	N/A
17	56	30	17
18	84	21	N/A
20	84	N/A	12
21	56	18	N/A
25	56	10	N/A
26	56	N/A	3

BDI scores range from 0-63, while PHQ-9 scores range from 0-27, with higher scores indicating more severe depressive symptoms. Sessions in which no scores were recorded are omitted.

Despite there being no FDA-approved treatment for TRBPD, esketamine was deemed appropriate for this patient due to her longstanding depressive symptoms with no episodes suggestive of mania or hypomania since May 2016. In addition, the patient began esketamine during a hospitalization for severe depression, thereby warranting a therapy that offers a swift clinical response, a trait esketamine is known for (17).

This patient’s improvement with esketamine following ECT nonresponse is in line with a retrospective study which found that those with treatment-resistant depression who did not respond to ECT were responsive to esketamine (18). While this retrospective study did not specifically look at TRBPD, it strengthens the premise that those who fail ECT may benefit from esketamine.

This is only the second case report in the existing literature pertaining to esketamine nasal spray being used to treat a patient with TRBPD. The first case report describes a patient being induced with esketamine twice weekly for one month, combined with venlafaxine, mirtazapine, lithium, and quetiapine. After noticeable improvement in the patient’s depression, he was put on a maintenance regimen lasting a year beginning once weekly, then once every two weeks, followed by once a month, and finally once every two months with an adjustment to his lithium dosage as result of a subhypomanic buying spree. However, his depression relapsed five months later and he was put back on his initial induction regimen. He subsequently improved and was placed on a maintenance plan once again, with the authors concluding that a monthly esketamine administration was recommended to ensure the patient remains in remission (19). What sets our report apart from the previous one is our inclusion of the dosage of esketamine administered. It is our hope that including this information will set a new standard for similar case reports in the future and address the need for esketamine studies that look at both frequency and dosage of treatment. Although both case reports show generally positive findings, they are limited in their generalizability. Therefore, it is imperative that more research is conducted, preferably randomized control trials that look at a diverse TRBPD patient population.

Another limitation in our case report is the inconsistent use of both BDI and PHQ-9 scores throughout the treatment. Neither were used in some sessions, including the first session, which complicated our ability to more objectively evaluate the patient’s clinical progress. Furthermore, it is unclear whether her post-relapse improvement can be attributed to the increase of the esketamine dosage to 84mg as it coincided with her aripiprazole being switched to lithium. This is a potential confounding variable that must be taken into account when evaluating esketamine’s effectiveness in this case.

Drawing from the uncertainty surrounding how the patient’s social stressors impacted the effectiveness of her esketamine treatment, future studies should gather data on their study population’s socioeconomic status, support system, education level, among other factors to more deeply assess how such factors can modulate esketamine outcomes. Lastly, there is still a need for studies that evaluate esketamine’s long term neurocognitive and cardiovascular side effects as well as the risk for tolerance.

## Clinical applications

A multitude of interventional psychiatric treatments have been developed in recent years with the purpose of ameliorating treatment-resistant depression. Transcranial magnetic stimulation (TMS), ECT, and ketamine/esketamine are the three that stand out for their level of evidence regarding safety and efficacy. ECT is the most efficacious but with the highest relative risk, TMS has the longest response durability with the lowest relative risk, while ketamine/esketamine has the fastest response (17). Although all are capable of aiding patients in achieving remission, the preference of one over the other is contingent upon factors such as the patient’s medical history, lifestyle, treatment preference, and ability to pay.

The fast resolution of depressive symptoms following ketamine/esketamine administration is appealing to clinicians and patients alike. This appeal was heightened by the FDA’s approval of intranasal esketamine for treatment-resistant depression with acute suicidal ideation or behavior in adults with major depressive disorder (MDD) (17). However, this approval does not extend to bipolar depression, as the clinical trials excluded participants with bipolar disorder (20). It can be argued that patients with a historic diagnosis of bipolar depression who have shown no recent signs of mania or hypomania can be included in the target population that this FDA approval was intended for, especially when considering that the literature to date shows minimal risk for manic or hypomanic switch (12). With that said, prior studies have consistently recommended that bipolar depression patients be put on a mood stabilizer prior to ketamine/esketamine infusions in order to further reduce any risk of mania or hypomania (10, 12).

## Data Availability

The original contributions presented in the study are included in the article/supplementary material. Further inquiries can be directed to the corresponding author.
