# Supplementary material for: Intranasal esketamine for ECT-refractory bipolar depression: a case report
Source: Front Psychiatry. 2026 Jul 30;17:1890002. doi: 10.3389/fpsyt.2026.1890002 (PMC13468001; doi:10.3389/fpsyt.2026.1890002)
Supplement: Supplementary file 1 [file DataSheet1.pdf]

Intranasal Ketamine for ECT-Refractory Bipolar Depression: A Case Report

Hackensack Meridian School of Medicine

Bruno Kallas (MS3), Max Garris (MS1), Dr. Muhammad Abbas MD

BACKGROUND

- Treatment-resistant bipolar depression is the failure to achieve a significant/sustained clinical response after 2+ approved and adequately dosed pharmacological treatments
- TRBPD currently has no FDA approved treatment.
- In the last decade, ketamine and esketamine have shown promise across multiple studies in the treatment of bipolar depression.
- Nevertheless, no clinical trials have been conducted yet with esketamine and bipolar depression, exposing the need for further research.

CASE REPORT

- This case report is about a 52 year old female with TRBPD who achieved remission for 3 months after receiving intranasal esketamine.
- This report adds to the growing esketamine literature by showing that a TRBPD patient can experience rapid benefit following esketamine therapy.

CASE PRESENTATION/RESULTS

PATIENT HISTORY

- 52 year old female with a 10 year history of TRBPD.
- Presented in March 2025 with one month of worsening depressive symptoms including anhedonia, insomnia, and suicidal ideation.
- Previous subtherapeutic response to SSRIs, atypical antidepressants, and antipsychotics.
- The patient had previously received ECT therapy for 5 months starting in March 2025 and discontinued in August 2025 due to subtherapeutic outcomes.

TREATMENT METHODS

- ECT initiated 03/26/25 for a total of 30 sessions, approx. 3x per week. RUL placement was used for the first 5 sessions with the remainder being bilateral.
- Intranasal esketamine was introduced 09/19/25 with a dosing of 56mg biweekly. Frequency was adjusted to once per week on 10/7/25, then every other week on 11/20/25.

ESKETAMINE RESULTS

- Esketamine nasal spray was introduced September 2025 and BDI scores remained <5 until December 2025.
- Patient related improvements in sleep duration and cognitive clarity, with minimal side effects.
- Relapse in depressive symptoms during December 2025, which patient attributed to interpersonal stressors.
- Patient expressed a desire to continue esketamine therapy and experienced a downward trend of BDI scores and depressive symptoms over the next few weeks.

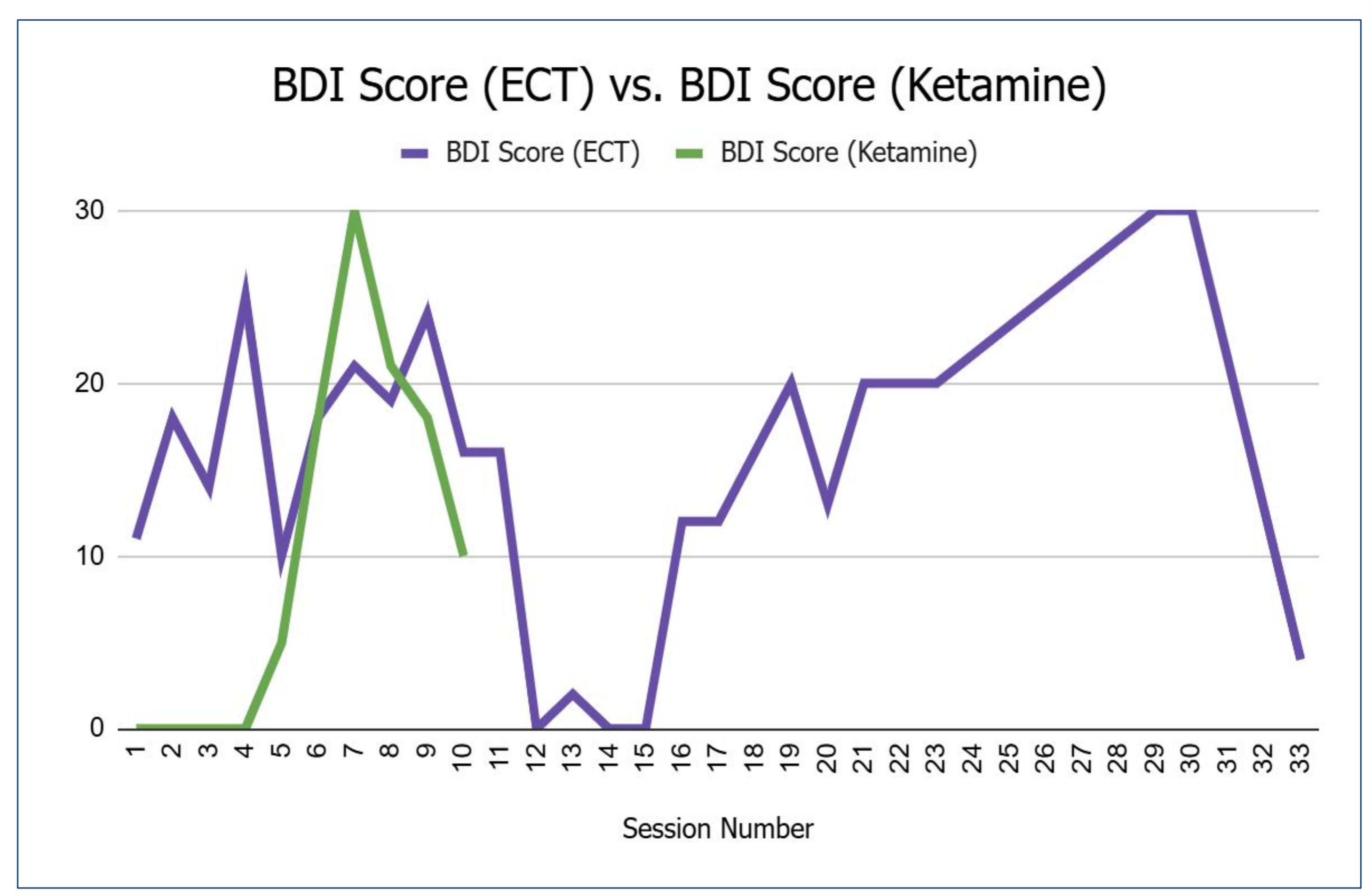

DISCUSSION / CONCLUSION

- This case exemplifies the therapeutic potential of esketamine therapy in patients with TRBPD.
- The long term impact of esketamine on this patient's bipolar depression is still unclear, as is the extent to which psychosocial stressors could interfere with her outcomes.
- This patient's success with esketamine aligns with a retrospective study which found that those with TRD who did not respond to ECT were responsive to esketamine.
- This is only the second case report in the existing literature pertaining to TRBPD treatment with esketamine nasal spray.
- Ultimately, further research is needed to elucidate generalizability for the TRBPD patient population.

REFERENCES

1) Fancy F., Rodrigues N.B., Di Vincenzo J.D., Chau E.H., Sethi R., Husain M.I., Gill H., Tabassum A., Mckenzie A., Phan L., et al. Real-world effectiveness of repeated ketamine infusions for treatment-resistant bipolar depression. Bipolar Disord. 2023;25:99–109. doi: 10.1111/bdi.13284.
2) Diaz AP, Fernandes BS, Quevedo J, Sanches M, Soares JC. Treatment-resistant bipolar depression: concepts and challenges for novel interventions. Brazilian Journal of Psychiatry. 2021;44(2). doi:https://doi.org/10.1590/1516-4446-2020-1627
3) Martinotti G, Dell'Osso B, Di Lorenzo G, et al. Treating bipolar depression with esketamine: Safety and effectiveness data from a naturalistic multicentric study on esketamine in bipolar versus unipolar treatment-resistant depression. Bipolar Disorders. Published online January 18, 2023. doi:https://doi.org/10.1111/bdi.13296
4) Skriptshak C, Reich A. Intranasal esketamine use in bipolar disorder: A case report. Mental Health Clinician. 2021;11(4):259-262. doi:https://doi.org/10.9740/mhc.2021.07.259
